# Role of gC1qR as a modulator of endothelial cell permeability and contributor to post-stroke inflammation and edema formation

**DOI:** 10.3389/fncel.2023.1123365

**Published:** 2023-06-13

**Authors:** Mychael Delgardo, Anthony J. Tang, Thilan Tudor, Andrés Pascual-Leone, E. Sander Connolly

**Affiliations:** Department of Neurological Surgery, Columbia University Irving Medical Center, New York, NY, United States

**Keywords:** stroke, edema, inflammation, endothelial cell permeability, complement

## Abstract

Ischemic stroke is a leading cause of death and disability worldwide. A serious risk of acute ischemic stroke (AIS) arises after the stroke event, due to inflammation and edema formation. Inflammation and edema in the brain are mediated by bradykinin, the formation of which is dependent upon a multi-ligand receptor protein called gC1qR. There are currently no preventive treatments for the secondary damage of AIS produced by inflammation and edema. This review aims to summarize recent research regarding the role of gC1qR in bradykinin formation, its role in inflammation and edema following ischemic injury, and potential therapeutic approaches to preventing post-stroke inflammation and edema formation.

## 1. Introduction

Despite advances in medicine and health care treatment and delivery, acute ischemic stroke (AIS) remains a leading cause of disability worldwide, with an estimated 795,000 strokes occurring every year in the US alone, 87% of which are ischemic (Tsao et al., 2022). Occlusion of the cerebral arteries that lead to AIS is accompanied by subsequent brain edema, which is mediated by bradykinin, suggesting that ischemic stroke is a thromboinflammatory disease. Furthermore, it has been established in preclinical models that gC1qR plays a central role in the assembly and formation of the biochemical pathway that leads to the generation of bradykinin and subsequent vascular leakage and associated inflammation (Fandaros et al., 2022).

Previous studies examining gC1qR have identified its presence in endothelial cells of the brain microvasculature and its role in the activation of the classical complement pathway (Yin et al., 2007). Although C1 inhibitors have been used as targeted therapies for edema formation following stroke (Weiss et al., 2020; Mercurio et al., 2021), the role gC1qR plays in edema formation post-stroke and its potential for being a therapeutic target have yet to be investigated. This paper aims to summarize recent research regarding gC1qR, particularly its role in bradykinin formation and, thus, a subsequent role in inflammation and edema development following ischemic stroke. We hope that with this review, we make a meaningful case that future research should investigate gC1qR as a potential therapeutic target in ameliorating post-stroke inflammation and edema formation.

## 2. gC1qR structure and its function in physiologic and pathophysiologic responses

gC1qR is a ubiquitously expressed, versatile binding protein that has been identified in multiple cellular compartments, including the mitochondrial matrix, cell surface, nucleus, and endoplasmic reticulum (Dembitzer et al., 2012). C1q, a complement component involved in the classical activation pathway, is a well-characterized binding partner of gC1qR, a receptor often described as a chaperone-like regulatory protein (Ghebrehiwet et al., 1994; Lynch et al., 1997; Rozanov et al., 2002). However, vitronectin (Lim et al., 1996), Factor XII, high molecular weight kininogen (HK) (Herwald et al., 1996), membrane type-1 metalloproteinase (MT1-MMP) (Rozanov et al., 2002), HIV Tat, and nuclear splicing factor 2 are ligands that also interact with this highly conserved protein and are involved in a diverse array of physiologic and pathophysiologic phenomena (Storz et al., 2000; Fogal et al., 2008; Fausther-Bovendo et al., 2010; Kim et al., 2011).

The structure of gC1qR is intimately connected to its functionality as a critical messenger in multiple signaling pathways. The 33 kDa protein is very acidic (pI = 4.15) and lacks traditional features of a cell surface receptor, such as a consensus motif for a transmembrane domain or a GPI anchor (Pednekar et al., 2016; Ghebrehiwet et al., 2019) gC1qR is composed of three monomers forming a trimer with a doughnut-shaped quaternary structure (Jiang et al., 1999). The receptor has seven anti-parallel β-strands filled by an N-terminal and two C-terminal α-helices, with an inner plasma-exposed surface highly negatively charged. In contrast, the membrane-facing outer ring is neutral or basic. The putative mechanism for cell-surface signaling via gC1qR involves the formation of associations with multiple transmembrane proteins and uses their respective signaling modalities to invoke specific functions (Fausther-Bovendo et al., 2010) gC1qR is unique in that it contains both ITAM and ITIM motifs, a rare structural consideration that reflects the binding protein’s dual ability to promote and inhibit cellular functions (Ghebrehiwet et al., 2019).

The involvement of gC1qR in the classical complement pathway is generally associated with its C1q binding activity. gC1qR is involved in classical pathway activation (Ghebrehiwet et al., 2006), through its interaction with the C1q subunit, which results in downstream generation of classical pathway products including C4d, C3b, and C5b-9 (Ghebrehiwet et al., 2006). This gC1qR-C1q-dependent activation of classical pathway products results in inflammatory peptide generation, cellular activation, and lysis (Fosbrink et al., 2005; Ghebrehiwet et al., 2006). More broadly, these complement-associated responses that stem from gC1qR-C1q binding form the basis of tissue destruction and cell death that are implicated in normal physiologic processes and the pathogenesis of inflammatory and infectious conditions.

Beyond complement activation, the intimate association of gC1qR with HK, bradykinin, and other peptides underlies normal physiologic responses, including vascular permeability and coagulation. The receptor protein has been implicated in coagulation through its association with thrombin and vitronectin (Lim et al., 1996; Pednekar et al., 2016). Binding of a vitronectin-thrombin-antithrombin complex to gC1qR, likely mediated through a specific gC1qR-vitronectin association, may form the basis of clearance of vitronectin-containing complexes in blood clots (Lim et al., 1996). C1q and gC1qR have been identified as mediators of dendritic cell development and may be involved in T-cell tolerance and anergy phenotypes (Kouser et al., 2015).

The localization of gC1qR in multiple cellular compartments and tissue types may explain its role in multiple pathophysiologic processes. gC1qR-positive cells generally localize to hypoxic or nutrient-deprived regions in tumor models, most notably in breast cancer, where the mitochondrial protein is overexpressed and located on the extracellular surface (Fogal et al., 2008; Song et al., 2019) gC1qR has also been implicated in atherosclerotic lesions, with a notable presence in activated macrophages (Peerschke et al., 2004; Song et al., 2019).

With respect to endothelial cell permeability, differential gC1qR expression is associated with shear stress in vessels due to blood flow (Yin et al., 2007; Fandaros et al., 2022) and may result in angioedema formation. gC1qR has also been implicated in bacterial and viral pathogenesis (Peerschke and Ghebrehiwet, 2007). Cell surface gC1qR is involved with *Listeria monocytogenes* virulence factor InlB, an activator of receptor tyrosine kinase Met (c-Met) that promotes bacterial dissemination (Braun et al., 2000; Shen et al., 2000; Peerschke and Ghebrehiwet, 2007). Inhibition of gC1qR-InlB binding has been shown to reduce L. monocytogenes invasion in a dose-dependent manner (Peerschke and Ghebrehiwet, 2007). In viral pathogenesis, gC1qR and its association with the Hepatitis C virus (HCV) core protein may induce T-cell suppression and result in a persistent infection (Yao et al., 2003).

## 3. Role of gC1qR in bradykinin formation and inflammatory disease

Besides binding the globular heads of C1q in the classical complement system, gC1qR also plays an integral part in the bradykinin formation. There are two general pathways for bradykinin formation (Kaplan et al., 2002). The one which involves gC1qR, the kinin-kallikrein pathway, will be the focus of this review.

**FIGURE 1:**
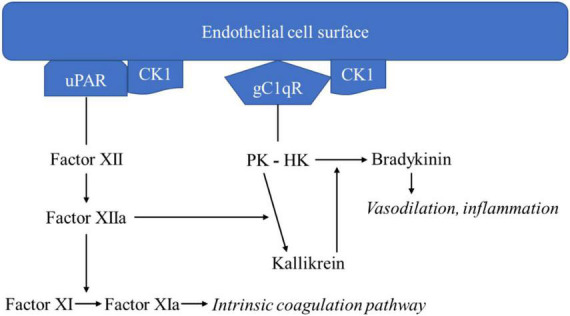
Kinin-kallikrein pathway leading to bradykinin formation.

The three major proteins in the kinin-kallikrein pathway are coagulation factor XII, prekallikrein (PK), and HK. Two bimolecular complexes–gC1qR-CK1 and uPAR-CK1–are organized on the endothelial cell surface and enable interactions among the proteins of the kinin-kallikrein pathway (Joseph et al., 2004). Factor XII is the initiating protein, and it attaches to uPAR-CK-1. PK is bound to HK, which is bound to the gC1qR component of the gC1qR-CK1 complex (Figure 1; Herwald et al., 1996).

Once Factor XII is bound to uPAR-CK-1, the kinin-kallikrein pathway auto-activation of factor XII to factor XIIa begins. Factor XIIa catalyzes two reactions: factor XI to factor XIa and PK to kallikrein. Factor XIa continues to initiate the intrinsic coagulation pathway. Kallikrein catalyzes the digestion of HK to form bradykinin. Bradykinin acts on bradykinin 1 and 2 receptors, inducing vasodilation and inflammatory responses. Kallikrein catalyzes further activation of factor XII to factor XIIa, exerting a positive feedback effect. Like bradykinin, kallikrein also has inflammatory properties, acting as a chemoattractant for neutrophils and monocytes, causing neutrophil aggregation and secretion of elastase, and activating factor B of the alternative complement pathway (Kaplan et al., 2002).

Given the crucial mediating role of gC1qR in the assembly of the kinin-kallikrein system, it is clear that gC1qR plays a role in bradykinin formation and inflammatory disease.

## 4. Involvement of gC1qR in brain edema development and other neurological disorders

Although the direct effect of gC1qR in post-ischemic brain edema has not been studied, it can be considered through studies of bradykinin.

Bradykinin is a potent vasodilator and pro-inflammatory agent in the body, produced in response to tissue injury. In the brain, bradykinin is linked with brain edema. In a study involving patients with traumatic brain injury and ischemic stroke, bradykinin levels in cerebrospinal fluid (CSF) samples were greater in the patient group than in the control group (Kunz et al., 2013). Additionally, CSF bradykinin concentration was positively associated with the extent of brain edema formation. Consequently, the role of gC1qR in bradykinin formation suggests that gC1qR may be indirectly involved in the development of post-ischemic brain edema.

The role of bradykinin in brain edema is elucidated by considering its functioning at the cellular level. Bradykinin binds to bradykinin one and bradykinin two receptors (B1R and B2R, respectively), leading to increased intracellular calcium release. Subsequently, claudin-5, a tight junction protein responsible for the integrity of the blood-brain barrier (BBB), is downregulated. As macromolecules enter the BBB, brain edema results (Gauberti et al., 2018).

Apart from affecting the BBB, bradykinin also contributes to brain edema by triggering pro-inflammatory signals in the cerebral circulation. Bradykinin promotes leukocyte rolling on the brain endothelium, induces mast cell and cyclo-oxygenase activation, and increases reactive oxygen species production in pial arterioles (Brian et al., 2001). The binding of bradykinin to B1R and B2R also attracts microglial cells to infarcted areas (Gauberti et al., 2018).

Of note, there remains controversy over the effects of bradykinin on its receptors. Some studies suggest B2R deficiency is protective against post-ischemic edema in the brain (Zausinger et al., 2002; Gröger et al., 2005; Su et al., 2009) while others report it has no effect or even exacerbates secondary brain injury after cerebral ischemia (Xia et al., 2006; Austinat et al., 2009). There also exists contradictory information on B1R. However, data regarding B1R are more homogeneous, and there is general agreement on its contribution to brain inflammation (Gauberti et al., 2018). To clarify the impact of bradykinin on post-ischemic brain edema, further investigation of B1R and B2R activity is needed. By considering the role of gC1qR in bradykinin formation and the actions of bradykinin in the brain, we highlight gC1qR as a potential target against post-ischemic brain edema, providing a more comprehensive understanding of the complex interplay between gC1qR and bradykinin signaling in this context.

The multifaceted roles of gC1qR in inflammation and edema, as well as its involvement in various signaling pathways, warrant further exploration in the context of neurological disease processes. Recent basic science studies have begun to elucidate the potential connections between gC1qR and neurological disorders such as Alzheimer’s disease. Alzheimer’s disease (AD) is characterized by chronic neuroinflammation, and recent findings suggest that gC1qR may play a role in this process through its association with the complement system and the complement systems association with AD. For example. Fonseca et al. (2009) observed complement activation in brains of a murine AD model, with the presence of complement proteins such as C1q, C3, and C4 in senile plaques and neurofibrillary tangles, which are hallmark features of the disease. Moreover, C1q, which binds to gC1qR, has been shown to bind to amyloid-beta (AB) peptide, which is a major component of the senile plaques in AD, and the binding of C1q to AB may trigger the complement cascade, resulting in inflammation and synaptic loss (Rogers et al., 1992; Hong et al., 2016). Although these findings do not directly implicate gC1qR in AD, they highlight the possible role of the complement system, including gC1qR-associated pathways, in the pathogenesis of the disease. Further research is needed to elucidate the specific role of gC1qR in AD and its potential as a therapeutic target.

In summary, both basic science and clinical studies have begun to uncover the connections between gC1qR’s role in inflammation and edema and the pathophysiology of various neurological diseases. Further research is needed to validate these findings and determine the therapeutic potential of targeting gC1qR in the context of neurological disorders.

## 5. Targeting gC1qR for therapy against post-stroke inflammation and brain edema

Targeting gC1qR as an approach to AIS management is an emergent area of interest based on the receptor’s proposed role in the pathogenesis of post-stroke inflammation. Given the high mortality rate of up to 80% associated with cerebral edema following AIS (Hacke et al., 1996; Berrouschot et al., 1998), novel complement-directed mechanisms of action are critical to multimodal treatment in the post-stroke setting. Multiple non-selective medical therapies, including osmotherapeutics, anesthetics, sedative agents, steroids, and induced hypothermia, have shown varying efficacy and safety in managing cerebral edema following stroke (Halstead and Geocadin, 2019).

In contrast to non-selective therapies, novel targeted therapeutics modulate components of cell-cell signaling pathways implicated in cerebral edema, including Sur1-Trpm4 channel inhibitors, vascular endothelial growth factor-related drugs, ion channel inhibitors, aquaporin blockade agents, and microRNAs (Halstead and Geocadin, 2019; Yao et al., 2020) gC1qR inhibition is a unique mechanism of action as it may attenuate pathologic inflammatory cascades by simultaneously mediating multiple pathways that contribute to cerebral edema. It has demonstrated promising results in pre-clinical models.

The specific interactions of therapeutic interest in the context of cerebral edema following AIS include the association of gC1qR with HK and gC1q. The gC1q-gC1qR interaction is mediated largely by the globular head A domain and less so by the globular heads B and C of the gC1q ligand (Ghebrehiwet et al., 2019). Concerning the receptor, the primary binding site of gC1q is located on residues 76–93, which is exposed on the plasma-facing side of the receptor (Ghebrehiwet et al., 2002). In terms of the HK-gC1qR relationship, a critical association that mediates inflammation and vascular permeability, structure-function studies involving gC1qR deletion mutants demonstrated that domains 190–202 and 204–218 on the receptor are involved in an HK binding pocket (Ghebrehiwet et al., 2011) that represents a prospective therapeutic target for post-AIS cerebral edema. Ghebrehiwet et al. (2011) also noted that the deletion of residues 154–162 of the gC1qR receptor and a single point mutation W233G significantly reduced bradykinin generation *in vitro*. This finding suggests that interactions outside of primary HK binding sites modulate the downstream generation of bradykinin. This potent pro-inflammatory peptide has been implicated in cerebral edema in AIS.

Monoclonal antibodies and peptide-based therapeutic approaches have been proposed in cancer therapy, infection, and general inflammation. The binding of HK to gC1qR is inhibited by monoclonal antibody (mAb) 74.5.2, directed against amino acids 204–218 of gC1qR, a site associated with the binding pocket for HK (Ghebrehiwet et al., 2011). Another HK binding site has been identified and confirmed at residues 190–202 (Ghebrehiwet et al., 2013), which was effectively blocked with mAbs 48 and 83 that were specific for this domain. A study focused on gC1qR-directed mAb therapy for mesothelioma indicated that mAb 60.11, directed against amino acids 76–93 of the C1q binding domain, significantly reduced tumor growth and aberrant angiogenesis *in vivo*. In contrast, mAb 74.5.2 had no effect (Peerschke et al., 2020). When administered together, mAbs 74.5.2 and 60.11 were associated with a significant reduction in *S. aureus* colonization of aortic valves, kidneys, and the spleen compared to untreated controls in a rat infective endocarditis model, suggesting that combination mAb therapy may be a viable approach (Peerschke et al., 2006). The authors suggest that gC1qR blockade with these two mAbs may modulate different functions of the receptor, with mAb 60.11 mediating direct complement activation and mAb 74.5.2 inhibiting the kinin/kallikrein system (Joseph et al., 1999; Peerschke et al., 2006). Given the synergistic roles of increased complement activity and kinin/kallikrein system activation in cerebral edema, these findings suggest that gC1qR targeting should be domain-specific to mediate these distinct proinflammatory pathways implicated in AIS.

Peptide-based approaches are of interest given some concerns surrounding mAb uptake and blood-brain barrier (BBB) permeability that are relevant for cerebral edema management. Domain 5 of HK, rich in histidine and arginine residues, contains a gC1qR interaction site along with HKH-20, a 20-amino acid peptide that is shown to mediate the interaction of HK with endothelial cells (Ghebrehiwet et al., 2013, 2019). Ghebrehiwet et al. (2019) provide evidence that a synthetic peptide directed to HKH-20 can inhibit HK-gC1qR binding *in vitro*, with the histidine residues critical for this interaction. There are unique considerations for gC1qR-directed peptide therapy for the CNS. The disulfide bonds in LyP-1, a tumor-homing peptide with gC1qR as its receptor, provide evidence of drug delivery challenges to the CNS. The disulfide linkages of LyP-1 are cleaved in the brain, hindering the targeting of glioma and metastatic brain tumors (Bickel et al., 1995). Further investigation into targeted peptide approaches for cerebral edema in AIS is essential and may help circumvent the current roadblocks to the therapeutic delivery of bulkier mAb-based alternatives.

Targeting ligands associated with nanoparticles represent a novel gC1qR-directed strategy that has been studied in the context of cancer treatment (Paasonen et al., 2016), using a mouse model of MCF10Ca1A breast tumor cells, identified a low molecular weight molecule with affinity for a tumor-homing binding site on gC1qR and attached it to an iron oxide nanoparticle. The resulting paramagnetic nanoparticles demonstrated robust homing to vasculature in MCF10Ca1A tumors *in vivo* compared to nanoparticles functionalized with a control peptide (Yenugonda et al., 2017) highlight the critical delivery challenges concerning the CNS; mAb and peptide-based therapies, the focus of many studies, do not readily cross the BBB, which poses a challenge to the treatment of AIS. Using a pharmacophore model for C1q and LyP-1, the authors identified a small molecule, M36, that could directly bind to gC1qR and inhibit glioma cell proliferation in culture. This approach provides *in vitro* evidence that a small molecule, instead of a peptide, may serve as the gC1qR homing molecule when associated with a nanoparticle (Yenugonda et al., 2017). A small molecule-based nanoparticle approach may be better suited to CNS delivery when compared to a larger peptide-homing agent when accounting for BBB permeability. For cerebral edema and drug delivery to the CNS, nanoparticles could avoid issues of proteolytic degradation, short-half life, and high volume of distribution associated with peptide-based drugs (Salameh and Banks, 2014), which limit delivery to the affected ischemic tissue.

gC1qR-directed therapy offers a novel mechanism of action that addresses the role of aberrant complement activation and generation of neuroinflammatory peptides in stroke pathogenesis. Complement inhibition has been shown to reduce the generation of downstream complement components such as C5a and attenuates pathologic microglial activation in stroke (Alawieh et al., 2018; Garred et al., 2021). Targeted therapy that inhibits the interaction between HK and gC1qR, such as the 74.5.2 mAb, has attenuated bradykinin-associated permeability changes in endothelial cells (Fandaros et al., 2022), a promising finding that offers a potential therapeutic target for post-stroke edema management.

Complement-directed therapies, particularly of early cascade components, can reduce downstream proinflammatory mediators and minimize inappropriate opsonization of cells that is associated with cell damage and impaired neuronal function (Garred et al., 2021). In contrast, existing non-targeted therapeutics for cerebral edema in AIS, such as hyperosmolar agents (e.g., mannitol, hypertonic saline) and induced hypothermia, rely on various mechanisms of action that operate substantially downstream from the inflammatory precedents of cerebral edema generation, such as osmotic gradient manipulation and metabolic demand reduction, to alleviate edema accumulation (Halstead and Geocadin, 2019). Targeted upstream approaches, including gC1qR-directed therapeutics, may be preferred due to direct attenuation of molecular signaling pathways (Fandaros et al., 2022) that underlie the vascular changes and proinflammatory factors that drive edema generation in AIS. Future investigations that explore gC1qR-directed therapies in the context of AIS will draw upon these established modalities and binding sites that have proven to be relevant to gC1qR and its role in generalized inflammation, vascular permeability, cancer, and infection.

## 6. Conclusion

We report the importance of gC1qR as a mediator of bradykinin formation, and consequently, a key receptor in inflammation and cerebral edema formation. We discuss in detail the involvement of gC1qR in the kinin-kallikrein bradykinin pathway, the role of bradykinin in edema following stroke, and important structure-function studies outlining the importance of gC1qR. We highlight the importance of mutation and monoclonal antibody studies against gC1qR that alter disease progression in animal models. Researchers focused on different conditions and different gC1qR-therapy modalities; however, gC1qR inhibition consistently was shown to ameliorate disease through anti-inflammatory mechanisms. Studies highlighted in this review suggest the potential viability of targeted gC1qR therapy through the attenuation of pathological inflammatory cascades. However, the direct application of targeted gC1qR inhibition on inflammation and edema formation following AIS remains to be evaluated. We suggest future studies optimize drug delivery through the BBB and assess the efficacy of gC1qR inhibition in an established AIS model.

## Author contributions

MD and EC contributed to the conception and design of the review manuscript. MD, TT, AT, and AP-L wrote the first draft of the manuscript. MD, TT, and AT contributed to the manuscript revision. All authors read and approved the final version of the manuscript.

## References

[B1] Alawieh A. Langley E. Tomlinson S. (2018). Targeted complement inhibition salvages stressed neurons and inhibits neuroinflammation after stroke in mice. *Sci. Transl. Med.* 10:eaao6459. 10.1126/scitranslmed.aao6459 29769288 PMC6689196

[B2] Austinat M. Braeuninger S. Pesquero J. Brede M. Bader M. Stoll G. (2009). Blockade of bradykinin receptor B1 but not bradykinin receptor B2 provides protection from cerebral infarction and brain edema. *Stroke* 40 285–293. 18988906 10.1161/STROKEAHA.108.526673

[B3] Berrouschot J. Sterker M. Bettin S. Köster J. Schneider D. (1998). Mortality of space-occupying (“malignant”) middle cerebral artery infarction under conservative intensive care. *Intensive Care Med.* 24 620–623. 9681786 10.1007/s001340050625

[B4] Bickel U. Kang Y. Pardridge W. (1995). In vivo cleavability of a disulfide-based chimeric opioid peptide in rat brain. *Bioconjug. Chem.* 6 211–218. 10.1021/bc00032a009 7599264

[B5] Braun L. Ghebrehiwet B. Cossart P. (2000). gC1q-R/p32, a C1q-binding protein, is a receptor for the InlB invasion protein of *Listeria monocytogenes*. *EMBO J.* 19 1458–1466. 10.1093/emboj/19.7.1458 10747014 PMC310215

[B6] Brian J. Faraci F. Moore S. (2001). COX-2-dependent delayed dilatation of cerebral arterioles in response to bradykinin. *Am. J. Physiol. Heart Circ. Physiol.* 280 H2023–H2029. 10.1152/ajpheart.2001.280.5.H2023 11299202

[B7] Dembitzer F. Kinoshita Y. Burstein D. Phelps R. Beasley M. Garcia R. (2012). gC1qR expression in normal and pathologic human tissues: Differential expression in tissues of epithelial and mesenchymal origin. *J. Histochem. Cytochem.* 60 467–474. 10.1369/0022155412440882 22638269 PMC3393077

[B8] Fandaros M. Joseph K. Kaplan A. Rubenstein D. Ghebrehiwet B. Yin W. (2022). gC1qR antibody can modulate endothelial cell permeability in angioedema. *Inflammation* 45 116–128. 10.1007/s10753-021-01532-w 34494203

[B9] Fausther-Bovendo H. Vieillard V. Sagan S. Bismuth G. Debré P. (2010). HIV gp41 engages gC1qR on CD4+ T cells to induce the expression of an NK ligand through the PIP3/H2O2 pathway. *PLoS Pathog.* 6:e1000975. 10.1371/journal.ppat.1000975 20617170 PMC2895652

[B10] Fogal V. Zhang L. Krajewski S. Ruoslahti E. (2008). Mitochondrial/cell-surface protein p32/gC1qR as a molecular target in tumor cells and tumor stroma. *Cancer Res.* 68 7210–7218. 10.1158/0008-5472.CAN-07-6752 18757437 PMC2562323

[B11] Fonseca M. Ager R. Chu S. Yazan O. Sanderson S. LaFerla F. (2009). Treatment with a C5aR antagonist decreases pathology and enhances behavioral performance in murine models of Alzheimer’s disease. *J. Immunol.* 183 1375–1383. 10.4049/jimmunol.0901005 19561098 PMC4067320

[B12] Fosbrink M. Niculescu F. Rus H. (2005). The role of c5b-9 terminal complement complex in activation of the cell cycle and transcription. *Immunol. Res.* 31 37–46. 15591621 10.1385/IR:31:1:37

[B13] Garred P. Tenner A. Mollnes T. (2021). Therapeutic targeting of the complement system: From rare diseases to pandemics. *Pharmacol. Rev.* 73 792–827. 33687995 10.1124/pharmrev.120.000072 PMC7956994

[B14] Gauberti M. Potzeha F. Vivien D. Martinez de Lizarrondo S. (2018). Impact of bradykinin generation during thrombolysis in ischemic stroke. *Front. Med.* 5:195. 10.3389/fmed.2018.00195 30018956 PMC6037726

[B15] Ghebrehiwet B. CebadaMora C. Tantral L. Jesty J. Peerschke E. (2006). gC1qR/p33 serves as a molecular bridge between the complement and contact activation systems and is an important catalyst in inflammation. *Adv. Exp. Med. Biol.* 586 95–105. 10.1007/0-387-34134-X_7 16893067

[B16] Ghebrehiwet B. Geisbrecht B. Xu X. Savitt A. Peerschke E. (2019). The C1q receptors: Focus on gC1qR/p33 (C1qBP, p32. HABP-1)1. *Semin. Immunol.* 45:101338. 10.1016/j.smim.2019.101338 31744753

[B17] Ghebrehiwet B. Jesty J. Peerschke E. (2002). gC1q-R/p33: Structure-function predictions from the crystal structure. *Immunobiology* 205 421–432. 10.1078/0171-2985-00143 12396004

[B18] Ghebrehiwet B. Jesty J. Vinayagasundaram R. Vinayagasundaram U. Ji Y. Valentino A. (2013). “Targeting gC1qR domains for therapy against infection and inflammation,” in *Complement therapeutics*, eds Lambris J. Holers V. Ricklin D. (New York, NY: Springer US), 97–110. 10.1007/978-1-4614-4118-2_6 23402021

[B19] Ghebrehiwet B. Jesty J. Xu S. Vinayagasundaram R. Vinayagasundaram U. Ji Y. (2011). Structure-function studies using deletion mutants identify domains of gC1qR/p33 as potential therapeutic targets for vascular permeability and inflammation. *Front. Immunol.* 2:58. 10.3389/fimmu.2011.00058 22282702 PMC3265123

[B20] Ghebrehiwet B. Lim B. Peerschke E. Willis A. Reid K. (1994). Isolation, cDNA cloning, and overexpression of a 33-kD cell surface glycoprotein that binds to the globular “heads” of C1q. *J. Exp. Med.* 179 1809–1821. 10.1084/jem.179.6.1809 8195709 PMC2191527

[B21] Gröger M. Lebesgue D. Pruneau D. Relton J. Kim S. Nussberger J. (2005). Release of bradykinin and expression of kinin B2 receptors in the brain: Role for cell death and brain edema formation after focal cerebral ischemia in mice. *J. Cereb. Blood Flow Metab.* 25 978–989. 10.1038/sj.jcbfm.9600096 15815587

[B22] Hacke W. Schwab S. Horn M. Spranger M. De Georgia M. von Kummer R. (1996). “Malignant” middle cerebral artery territory infarction: Clinical course and prognostic signs. *Arch. Neurol.* 53 309–315. 10.1001/archneur.1996.00550040037012 8929152

[B23] Halstead M. Geocadin R. (2019). The medical management of cerebral edema: Past, present, and future therapies. *Neurotherapeutics* 16 1133–1148. 31512062 10.1007/s13311-019-00779-4 PMC6985348

[B24] Herwald H. Dedio J. Kellner R. Loos M. Müller-Esterl W. (1996). Isolation and characterization of the kininogen-binding protein p33 from endothelial cells: Identity with the gC1q receptor. *J. Biol. Chem.* 271 13040–13047. 10.1074/jbc.271.22.13040 8662673

[B25] Hong S. Beja-Glasser V. Nfonoyim B. Frouin A. Li S. Ramakrishnan S. (2016). Complement and microglia mediate early synapse loss in Alzheimer mouse models. *Science* 352 712–716. 27033548 10.1126/science.aad8373 PMC5094372

[B26] Jiang J. Zhang Y. Krainer A. Xu R. (1999). Crystal structure of human p32, a doughnut-shaped acidic mitochondrial matrix protein. *Proc. Natl. Acad. Sci. U.S.A.* 96 3572–3577. 10.1073/pnas.96.7.3572 10097078 PMC22335

[B27] Joseph K. Ghebrehiwet B. Kaplan A. (1999). Cytokeratin 1 and gC1qR mediate high molecular weight kininogen binding to endothelial cells. *Clin. Immunol.* 92 246–255. 10479529 10.1006/clim.1999.4753

[B28] Joseph K. Tholanikunnel B. Ghebrehiwet B. Kaplan A. (2004). Interaction of high molecular weight kininogen binding proteins on endothelial cells. *Thromb. Haemost.* 91 61–70. 14691569 10.1160/TH03-07-0471

[B29] Kaplan A. Joseph K. Silverberg M. (2002). Pathways for bradykinin formation and inflammatory disease. *J. Allergy Clin. Immunol.* 109 195–209. 11842287 10.1067/mai.2002.121316

[B30] Kim K. -B. Yi J. -S. Nguyen N. Lee J. -H. Kwon Y. -C. Ahn B. -Y. (2011). Cell-surface receptor for complement component C1q (gC1qR) is a key regulator for lamellipodia formation and cancer metastasis. *J. Biol. Chem.* 286 23093–23101. 10.1074/jbc.M111.233304 21536672 PMC3123076

[B31] Kouser L. Madhukaran S. Shastri A. Saraon A. Ferluga J. Al-Mozaini M. (2015). Emerging and novel functions of complement protein C1q. *Front. Immunol.* 6:317. 10.3389/fimmu.2015.00317 26175731 PMC4484229

[B32] Kunz M. Nussberger J. Holtmannspötter M. Bitterling H. Plesnila N. Zausinger S. (2013). Bradykinin in blood and cerebrospinal fluid after acute cerebral lesions: Correlations with cerebral edema and intracranial pressure. *J. Neurotrauma* 30 1638–1644. 10.1089/neu.2012.2774 23638655

[B33] Lim B. Reid K. Ghebrehiwet B. Peerschke E. Leigh L. Preissner K. (1996). The binding protein for globular heads of complement C1q, gC1qR. Functional expression and characterization as a novel vitronectin binding factor. *J. Biol. Chem.* 271 26739–26744. 10.1074/jbc.271.43.26739 8900153

[B34] Lynch N. J. Reid K. B. van den Berg R. Daha M. R. Leigh L. A. Ghebrehiwet B. (1997). Characterisation of the rat and mouse homologues of gC1qBP, a 33 kDa glycoprotein that binds to the globular “heads” of C1q. *FEBS Lett.* 418 111–114. 10.1016/s0014-5793(97)01348-3 9414106

[B35] Mercurio D. Piotti A. Valente A. Oggioni M. Ponstein Y. Van Amersfoort E. (2021). Plasma-derived and recombinant C1 esterase inhibitor: Binding profiles and neuroprotective properties in brain ischemia/reperfusion injury. *Brain Behav. Immun.* 93 299–311. 10.1016/j.bbi.2021.01.002 33444732

[B36] Paasonen L. Sharma S. Braun G. Kotamraju V. Chung T. She Z. (2016). New p32/gC1qR ligands for targeted tumor drug delivery. *Chembiochem* 17 570–575. 10.1002/cbic.201500564 26895508 PMC5433940

[B37] Pednekar L. Pathan A. Paudyal B. Tsolaki A. Kaur A. Abozaid S. (2016). Analysis of the interaction between globular head modules of human C1q and its candidate receptor gC1qR. *Front. Immunol.* 7:567. 10.3389/fimmu.2016.00567 28018340 PMC5153404

[B38] Peerschke E. Ghebrehiwet B. (2007). The contribution of gC1qR/p33 in infection and inflammation. *Immunobiology* 212 333–342. 17544818 10.1016/j.imbio.2006.11.011 PMC2001281

[B39] Peerschke E. Bayer A. Ghebrehiwet B. Xiong Y. (2006). gC1qR/p33 blockade reduces *Staphylococcus aureus* colonization of target tissues in an animal model of infective endocarditis. *Infect. Immun.* 74 4418–4423. 10.1128/IAI.01794-05 16861627 PMC1539591

[B40] Peerschke E. Minta J. Zhou S. Bini A. Gotlieb A. Colman R. (2004). Expression of gC1q-R/p33 and its major ligands in human atherosclerotic lesions. *Mol. Immunol.* 41 759–766. 10.1016/j.molimm.2004.04.020 15234555

[B41] Peerschke E. Stier K. Li X. Kandov E. de Stanchina E. Chang Q. (2020). gC1qR/HABP1/p32 is a potential new therapeutic target against mesothelioma. *Front. Oncol.* 10:1413. 10.3389/fonc.2020.01413 32903438 PMC7435067

[B42] Rogers J. Cooper N. Webster S. Schultz J. McGeer P. Styren S. (1992). Complement activation by beta-amyloid in Alzheimer disease. *Proc. Natl. Acad. Sci. U.S.A.* 89 10016–10020. 1438191 10.1073/pnas.89.21.10016 PMC50268

[B43] Rozanov D. Ghebrehiwet B. Ratnikov B. Monosov E. Deryugina E. Strongin A. (2002). The cytoplasmic tail peptide sequence of membrane type-1 matrix metalloproteinase (MT1-MMP) directly binds to gC1qR, a compartment-specific chaperone-like regulatory protein. *FEBS Lett.* 527 51–57. 10.1016/s0014-5793(02)03153-8 12220632

[B44] Salameh T. Banks W. (2014). Delivery of therapeutic peptides and proteins to the CNS. *Adv. Pharmacol.* 71 277–299. 10.1016/bs.apha.2014.06.004 25307220 PMC6087545

[B45] Shen Y. Naujokas M. Park M. Ireton K. (2000). InIB-dependent internalization of *Listeria* is mediated by the Met receptor tyrosine kinase. *Cell* 103 501–510. 10.1016/s0092-8674(00)00141-0 11081636

[B46] Song N. Zhao L. Zhu M. Zhao J. (2019). Recent progress in LyP-1-based strategies for targeted imaging and therapy. *Drug Deliv.* 26 363–375. 10.1080/10717544.2019.1587047 30905205 PMC6442157

[B47] Storz P. Hausser A. Link G. Dedio J. Ghebrehiwet B. Pfizenmaier K. (2000). Protein kinase C [micro] is regulated by the multifunctional chaperon protein p32. *J. Biol. Chem.* 275 24601–24607. 10.1074/jbc.M002964200 10831594

[B48] Su J. Cui M. Tang Y. Zhou H. Liu L. Dong Q. (2009). Blockade of bradykinin B2 receptor more effectively reduces postischemic blood–brain barrier disruption and cytokines release than B1 receptor inhibition. *Biochem. Biophys. Res. Commun.* 388 205–211. 10.1016/j.bbrc.2009.07.135 19647718

[B49] Tsao C. Aday A. Almarzooq Z. Alonso A. Beaton A. Bittencourt M. (2022). Heart disease and stroke statistics—2022 update: A report from the American Heart Association. *Circulation* 145 e153–e639. 35078371 10.1161/CIR.0000000000001052

[B50] Weiss E. Dhir T. Collett A. Reola M. Kaplan M. Minimo C. (2020). Effect of complement C1-esterase inhibitor on brain edema and inflammation after mild traumatic brain injury in an animal model. *Clin. Exp. Emerg. Med.* 7 87–94. 10.15441/ceem.19.050 32635699 PMC7348678

[B51] Xia C. Smith R. Jr. Shen B. Yang Z. Borlongan C. Chao L. (2006). Postischemic brain injury is exacerbated in mice lacking the kinin B2 receptor. *Hypertension* 47 752–761. 10.1161/01.HYP.0000214867.35632.0e 16534002

[B52] Yao Y. Zhang Y. Liao X. Yang R. Lei Y. Luo J. (2020). Potential therapies for cerebral edema after ischemic stroke: A mini review. *Front. Aging Neurosci.* 12:618819. 10.3389/fnagi.2020.618819 33613264 PMC7890111

[B53] Yao Z. Eisen-Vandervelde A. Ray S. Hahn Y. (2003). HCV core/gC1qR interaction arrests T cell cycle progression through stabilization of the cell cycle inhibitor p27Kip1. *Virology* 314 271–282. 10.1016/s0042-6822(03)00419-7 14517080

[B54] Yenugonda V. Nomura N. Kouznetsova V. Tsigelny I. Fogal V. Nurmemmedov E. (2017). A novel small molecule inhibitor of p32 mitochondrial protein overexpressed in glioma. *J. Transl. Med.* 15:210. 10.1186/s12967-017-1312-7 29047383 PMC5648515

[B55] Yin W. Ghebrehiwet B. Weksler B. Peerschke E. (2007). Classical pathway complement activation on human endothelial cells. *Mol. Immunol.* 44 2228–2234. 17173972 10.1016/j.molimm.2006.11.012 PMC1865514

[B56] Zausinger S. Lumenta D. Pruneau D. Schmid-Elsaesser R. Plesnila N. Baethmann A. (2002). Effects of LF 16-0687 Ms, a bradykinin B(2) receptor antagonist, on brain edema formation and tissue damage in a rat model of temporary focal cerebral ischemia. *Brain Res.* 950 268–278. 10.1016/s0006-8993(02)03053-6 12231253

